# Arteriovenous malformation and dementia: A case report

**DOI:** 10.1590/S1980-5764-2016DN1003012

**Published:** 2016

**Authors:** Sonia Maria Dozzi Brucki, Samila Marissa Pinheiro Gomes

**Affiliations:** 1 MD, PhD, Hospital Santa Marcelina, São Paulo SP, Brazil.; 2 MD, Resident of Neurology, Hospital Santa Marcelina, São Paulo SP, Brazil.

**Keywords:** dementia, arteriovenous malformation, cognition

## Abstract

Arteriovenous malformation (AVM) is a congenital lesion commonly associated with intracranial hemorrhage. We describe a woman with an AVM (Spetzler-Martin grade V) and presenile progressive dementia. The association between AVM and cognition is discussed.

## RESUMO

Malformação arteriovenosa (MAV) é uma lesão congênita comumente associada à hemorragia intracraniana. Nós descrevemos uma mulher com uma MAV (Spetzler-Martin grau V) com uma demência pré-senil progressiva. Será discutida a associação entre MAV e cognição.

## INTRODUCTION

Arteriovenous malformation (AVM) has a congenital origin and is composed of a tangle of arteries and veins connected by fistulae, where the central part is a nidus. The most common symptom is hemorrhage, but other symptoms include headache, seizures, stroke-like symptoms, and ischemic stroke. Cognitive findings are rarely reported in the literature, with some descriptions of a progressive course among case series reports.

We described a 63-year-old illiterate housewife with a history of right motor partial seizures and generalization since the age of 23 years. In July of 2013 she sought medical care at the Emergency Room due to acute right hemiparesis; during the investigation a predominantly left temporoparietal AVM was disclosed (classified as Spetzler-Martin grade V considering: > 6 cm, eloquent area, superficial and deep). At the time, the treatment elected was conservative with no indication for surgery, endovascular embolization or radiosurgery.^1^ ^,^ ^2^

The husband reported she had a three-year history of progressive difficulties in instrumental activities of daily living, such as cooking and housekeeping, shopping and financial control. Concomitantly, he observed that she presented difficulties in the acquisition of new information, and exhibited topographic and temporal disorientation. Cognition and functioning progressively declined with partial dependency for basic activities of daily living (bathing, grooming and clothing); apathy was the predominant neuropsychiatric symptom (without aggression, irritability, or sleep problems). There was no family history of dementia. She was in use of medication for seizure control (carbamazepine 600 mg/d and phenobarbital 100 mg/d). 

Neurological examination revealed right hemiparesis, right tactile and pain hypoesthesia, poor fluency, temporal and spatial disorientation, and a Mini-Mental State Examination score of 5 points (one for immediate memory, two for naming, one for repetition, and one for commands).

**Figure 1:**
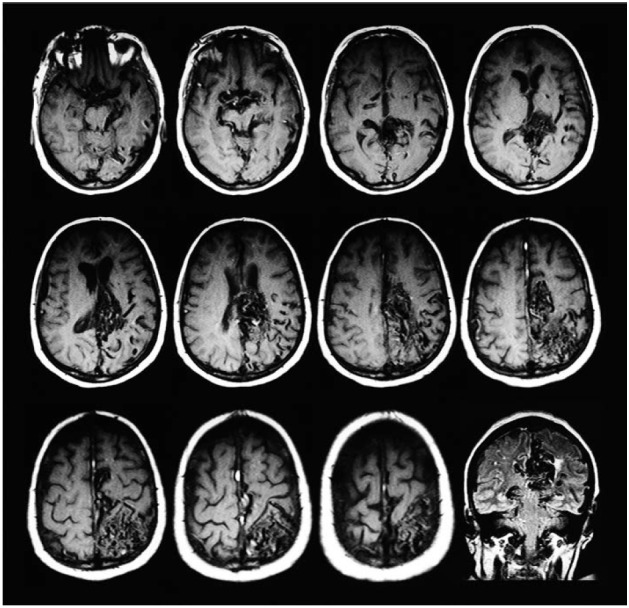
MRI T1-weighted images showing multiple flow voids and engorgement of arteries and veins (axial slices) and Flair (coronal slice).

The MRI performed in 2016 showed a massive AVM in the left hemisphere of the frontotemporoparietal region (9.2 × 6.0 cm) with parenchymal compression and microangiopathy. There were many low-signal spots within or around the mass on T1, T2, and fluid-attenuated inversion recovery (FLAIR) sequences: the "flow voids" of feeding arteries with ectasia (anterior, middle and posterior arteries) and draining veins (with drainage by left Trolard and Rosenthal veins). There was no sign of hemorrhage (Figure 1).

Her EEG showed diffuse disorganization and acute waves in the left temporal region. A diagnosis of dementia caused by AVM was defined (chronic hypoperfusion and space-occupying lesion). 

## DISCUSSION

In the literature there are scant data on cognitive deficits observed in AVM patients. Cognition is described in terms of neurological deficit or using a weak definition of dementia. We used a well-defined criteria of dementia based on multiple cognitive impairments, behavioral disturbance, and difficulties in activities of daily living. 

Cognitive impairment was observed in a case series of 43 patients by Olivecrona & Riives in 1948; where they had reported "mental impairment/deterioration" or "memory deterioration" in 11 individuals.^3^

AVMs cause neurological dysfunction through several mechanisms such as hemorrhage in subarachnoid space, intraventricular or intralobar hematoma, seizures, and progressive neurological deficit.^4^

In our case, tangles of arteries and veins caused major compromise of the left cerebral hemisphere, involving the thalamus, hippocampus, posterior cingulate gyrus, precuneus and temporo-parietal junction. These areas are part of the limbic system and default mode network, both heavily involved in cognition. 

Focal neurological deficit can be the first sign in 5% to 15% of AVM in hospital-based studies but are relatively rare. In a large sample, 7% of 735 untreated AVM presented with focal neurological deficit; with four possible outcomes: stable, progressive, fluctuating or reversible. Among these focal deficits, cognition was compromised in 10 out of 53 cases and progressive in four patients.^5^ In another cohort of 343 patients, only two individuals had dementia.^6^ The cause of these symptoms has rarely been investigated but possible mechanisms are mechanical displacement of brain tissue (mass effect), compressive effect of venous dilation, and neuronal loss due to chronic hypoperfusion.^5^ The relative rarity of focal neurological signs could be explained by chronic mass lesions and chronic hypoperfusion in association with compensational mechanisms, such as remote neuronal activation and reorganization of cerebral function.^5^ ^,^ ^7^ ^,^ ^8^ In a review concerning neuroplasticity, there were scant data on neuroplasticity in patients with grade V - AVM (Spetzler-Martin grade).^8^

There are more reported cases associating cognitive impairment to dural arteriovenous fistula, as part of AVMs; these cases have shown frontal lobe dysfunction; temporal, spatial and personal disorientation and in calculus, with rapidly progressive cognitive symptoms and partial recuperation after endovascular treatment.^9^ ^,^ ^10^

Our patient may have had a low pre-morbid cognitive level since AVM developed throughout her life and she is illiterate with an undemanding activity. Functional decline was only noted in the past few years, evolving to overt dementia. Although her history reflects a degenerative condition with chronic and progressive involvement of cognition and function, we observed a consistent cause of disability with no hippocampal atrophy, presenile onset with no family history, and a previous poor background. The patient presented a rare manifestation (dementia) of a massive AVM, where focal motor and sensory deficits are relatively scarce in reported series. 
